# Determining the Minimal Number of Passive Hip and Knee Joint Movement Repetitions Recommended for the Stiff Rectus Femoris Muscle Due to Osgood–Schlatter Disease

**DOI:** 10.3390/children13040460

**Published:** 2026-03-27

**Authors:** Naoki Ikeda, Ayumi Yoshikawa, Shota Yamaguchi, Takuya Nishioka, Genko Karasawa, Takayuki Inami

**Affiliations:** 1 Institute of Physical Education, Keio University, 4-1-1 Hiyoshi, Yokohama 223-8521, Japan; nikeda305@keio.jp (N.I.); ayumi.y@keio.jp (A.Y.); yamaguchi.s@keio.jp (S.Y.); nishioka.t@keio.jp (T.N.); 2 Karasawa Orthopedic Clinic, 22-1 Toyooka, Yokohama 230-0062, Japan; gkarasawa@yahoo.co.jp

**Keywords:** shear wave elastography, shear modulus, acute effect, muscle elasticity, manual therapy

## Abstract

Background/Objectives: Osgood–Schlatter disease (OSD) is a common overuse condition in adolescents characterized by increased stiffness of the rectus femoris muscle, which contributes to pain and functional limitations around the knee. We investigated whether repeating 10 min passive joint movements of the hip and knee produces additional immediate reductions in elevated rectus femoris (RF) stiffness in adolescents with OSD. Methods: Fifteen patients (10–14 years of age) diagnosed with bilateral OSD were included. The legs of the participants were randomly assigned to either the intervention or the non-intervention side (control). The intervention side received two sets of 10 min of passive joint movement to the hip and knee, while the control side rested. RF stiffness was measured before the intervention and immediately after one and two sets of passive joint movements. Results: On the intervention side, RF stiffness decreased significantly from pre to post-1 and from pre to post-2; however, RF stiffness did not differ significantly between post-1 and post-2. None of the parameters changed significantly on the control side (rest condition). Conclusions: Passive joint exercise beyond one repetition (one set for 10 min) did not result in a further decrease in RF stiffness and is likely unnecessary for RF muscle stiffness due to OSD.

## 1. Introduction

Osgood–Schlatter disease (OSD) is common among physically active adolescents and is characterized by traction apophysitis of the tibial tuberosity [1,2]. Previous studies have reported that increased tension in the quadriceps femoris is a risk factor for OSD [2,3]. Furthermore, several studies have assessed rectus femoris (RF) stiffness using shear-wave elastography to evaluate the pathophysiology of OSD [4,5].

A recent study proposed passive joint movement of the hip and knee as a physical therapy to alleviate OSD symptoms [5]. This technique involves therapist-applied passive movements at the hip and knee performed rhythmically (approximately 1 Hz) with multiplanar components, as detailed in the protocol below. A previous study reported that a 10 min session of passive joint movement reduced RF stiffness and improved outcomes of the heel–buttock distance (HBD) test and knee subjective pain in patients with OSD [5], highlighting the effectiveness of passive joint movement in OSD. However, it remains unclear whether repeating this intervention beyond a single 10 min set would produce additional benefits.

When muscles and tendons are continuously exposed to mechanical stimulation during physical therapy, such as static stretching, their viscoelasticity (i.e., decreased stiffness) changes [6,7]. Furthermore, in healthy individuals, a meta-analysis review showed that the longer the stretching duration, the greater the decrease in muscle-tendon unit (MTU) stiffness [6]. However, these soft tissue adaptations slow down because of structural and neural constraints beyond a certain threshold. As stretching sets are repeated (i.e., the duration of the stretches increases), changes in MTU stiffness and joint flexibility, which are affected by MTU stiffness, also plateau [8,9,10]. Therefore, passive joint movement may show a similar time course of stiffness reduction to other mechanically applied therapies, although this has not been established in OSD. However, since patients with OSD exhibit higher RF stiffness than healthy individuals, it may take more than a single 10 min set to reach a plateau in muscle stiffness.

The purpose of this study was to investigate whether repeating two sets of 10 min passive joint movements of the hip and knee would enhance the reduction in RF stiffness in patients with OSD. We hypothesized that performing two sets of 10 min passive joint movements of the hip and knee would produce a greater reduction in RF stiffness associated with OSD than a single set.

## 2. Materials and Methods

### 2.1. Participants

This study included 15 patients diagnosed with bilateral OSD (12 male: age 13 ± 1 years, height 152.5 ± 9.1 cm, weight 45.1 ± 10.7 kg; 3 female: age 11 ± 2 years, height 151.0 ± 7.0 cm, weight 42.4 ± 3.7 kg; mean ± standard deviation). All participants participated in sports activities (volleyball [*n* = 3], soccer [*n* = 3], baseball [*n* = 2], basketball [*n* = 2], gymnastics [*n* = 2], track and field [*n* = 1], badminton [*n* = 1], and ice hockey [*n* = 1]) for a mean of 2–3 sessions per week. Informed consent was obtained from all participants after they were provided with details of the study’s purpose, procedures, benefits, and risks. This study was reviewed and approved by the Ethical Review Committee for Human Subjects Research of our institution (Approval No.: 23-004).

### 2.2. Study Design

This study aimed to investigate the effects of passive joint movement on RF stiffness and immediate changes in stiffness following repeated sets. For each participant, one leg was randomly assigned to the passive joint movement condition and the contralateral leg to the control (rest) condition (15 intervention legs; 15 control legs). Both legs were measured in all participants; one leg underwent the passive joint movement intervention, and the contralateral leg served as the control condition without passive movement. The diagnosis of OSD, assessment of muscle stiffness, HBD test, and passive joint movement intervention were conducted on the same day. RF stiffness was measured at pre, immediately after set 1 (post-1), and immediately after set 2 (post-2) for both the intervention and control sides. The HBD test was performed twice: prior to the intervention and immediately following the second set (post 2), and was not measured after the first set.

### 2.3. Diagnosis of OSD

Based on previous studies, OSD was diagnosed using ultrasonography (Sonimage MX1; Konica Minolta Inc., Tokyo, Japan) [5,11]. An orthopedic surgeon evaluated the pain during movement, prominence of the tibial tuberosity, and tenderness. OSD was defined as the presence of one or more of these clinical findings, in combination with ultrasonographic evidence of either a loose bone fragment or cartilaginous swelling at the tibial tuberosity. A loose bone fragment was defined as a hyperechoic structure observed within the cartilage surrounded by hypoechoic tissue, except for the secondary ossification center. Cartilaginous swelling was defined as a skin protrusion caused by the elevation of the underlying cartilage. During the ultrasonographic examination, the participants maintained the knee joint at 90° of flexion, and a linear ultrasound probe was placed over the tibial tuberosity. The tibial tuberosity was evaluated using longitudinal ultrasound images.

### 2.4. Passive Joint Movement Protocol

**Figure 1:**
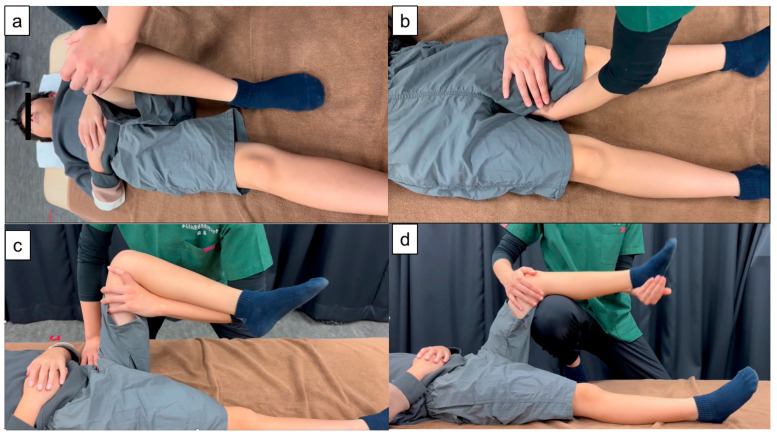
Passive joint movement ((**a**): stage 1; (**b**): stage 2; (**c**): stage 3; (**d**): stage 4).

Passive joint movement was performed in a controlled environment maintained at 24 °C, with the participants positioned supine on a bed. Following the protocol of a previous study [5], participants rested for 10 min before the intervention. The passive joint movement intervention comprised two 10 min sets of therapist-applied passive movements of the hip and knee, separated by a 3 min interval. Each 10 min set consisted of four stages applied in sequence. In stage 1, the therapist passively moved the hip through approximately 0–45° of adduction and abduction with the knee maintained at 90° of flexion (Figure 1a). In stage 2, the therapist passively moved the hip through approximately 0–45° of internal and external rotation with the knee maintained at 10° of flexion (Figure 1b). In stage 3, the therapist passively extended the hip from approximately 90° of flexion toward 0–30° (up to 45° as tolerated), while minimizing anterior pelvic tilt (Figure 1c). In stage 4, the therapist passively extended the knee from 90° of flexion to 45° of flexion (Figure 1d). A metronome (50–60 cycles/min; approximately 1 Hz) was used to standardize the movement rhythm. Each stage lasted 2.5 min (total: 10 min per set). All interventions were delivered by two physical therapists with more than five years of clinical experience and were performed within a submaximal, pain-free range. On the control side (contralateral limb), participants rested for an equivalent duration without passive joint movement.

### 2.5. Measurement of RF Muscle Stiffness

**Figure 2:**
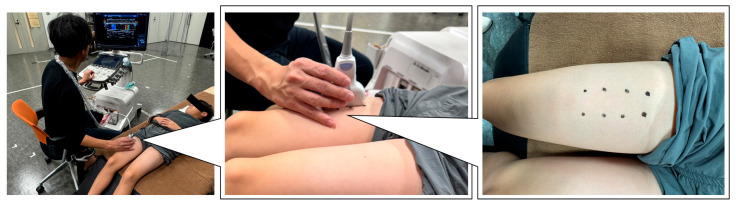
Illustration of the measurement of the rectus femoris muscle stiffness.

RF stiffness was quantified as the RF shear modulus measured using shear-wave elastography (Aplio 300; Canon Medical Systems Corporation, Tochigi, Japan). In accordance with previous studies [4,5], the participants were positioned supine on a bed during the measurement, with the knee joint maintained at 45° of flexion (0° defined as full extension) (Figure 2).

The measurement site for the RF was set at the midpoint of the muscle belly, which is located at approximately 50% of the proximal thigh length. A linear ultrasound probe was placed over the measurement site to acquire longitudinal B-mode images with color mapping, representing the shear modulus of the RF. Before image acquisition, the stability of the color mapping was confirmed for several seconds, after which three consecutive images were captured. The participants were instructed to relax during the measurements.

**Figure 3:**
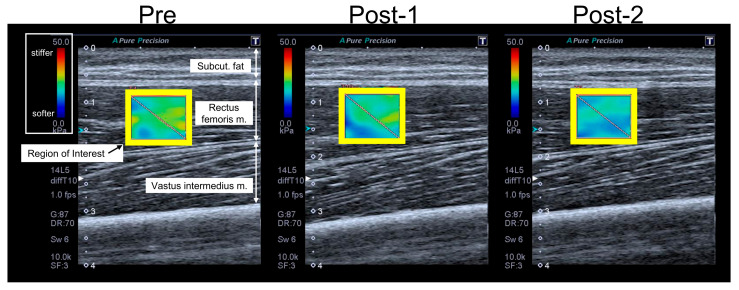
An example of shear wave elastography imaging. Analysis of muscle shear modulus, with regions of interest (ROIs) indicated by yellow squares.

The acquired images were analyzed using the ultrasound device software built into the system. A rectangular region of interest (ROI) was marked within each image, and the mean shear modulus within the ROI was recorded (Figure 3). The muscle stiffness of the RF was defined as the average shear modulus obtained from the three images. All ultrasound measurements and analyses were performed by an experienced examiner with >20 years of clinical experience.

### 2.6. Measurement of Heel–Buttock Distance (HBD)

**Figure 4:**
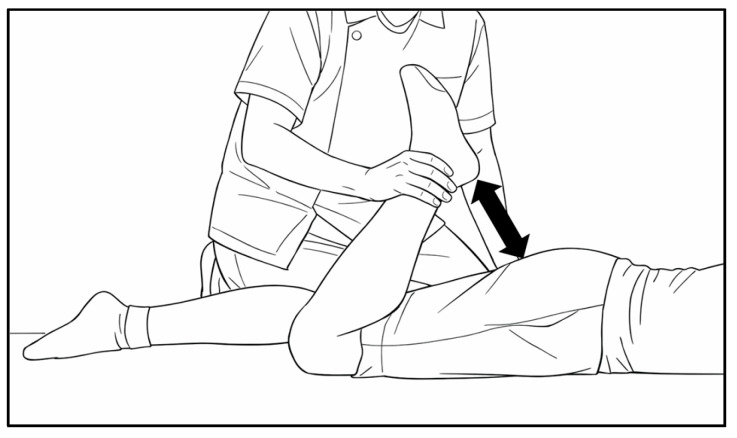
Illustration of the measurement during the heel–buttock distance test.

Based on previous studies [3,5], HBD was measured as an indicator of quadriceps muscle tightness and soreness (Figure 4). The participants were positioned prone on a bed with both lower limbs aligned. The examiner grasped the ankle on one side and passively flexed the knee until the participant reached a tolerable limit of knee flexion. HBD was defined as the distance between the heel and buttocks, with smaller values indicating improved quadriceps flexibility.

### 2.7. Statistical Analysis

All data are presented as the mean ± standard deviation (SD). A two-way repeated-measures ANOVA [condition × time (pre, post 1, post 2)] was conducted for RF stiffness. For HBD, which was measured at pre and post 2 only, a two-way repeated-measures ANOVA [condition × time (pre, post 2)] was conducted using the statistical software IBM SPSS Statistics (version 29, IBM Corp., Armonk, NY, USA). When a significant interaction or main effect of time was observed, Bonferroni post hoc tests were performed for each condition. Cohen’s d (post hoc comparison) and partial η^2^ (η_p_^2^: ANOVA) were used to calculate the effect size. Cohen’s d was obtained using the following equation:

$$d=\left(\frac{M_{\mathrm{diff}}}{SD_{\mathrm{pooled}}}\right)\times \sqrt{\left(2\left(1-r\right)\right)}$$

where M_diff_ is the difference between the mean values of the pre- and post-measurements, and r is the correlation between the mean values [12]. The effect size was defined as follows: 0.20–0.50 small effect, 0.50–0.80 medium effect, and ≥0.80 large effect [13]. A priori statistical power analysis indicated that this study design would require 14 participants per condition (repeated-measures ANOVA within factors; effect size, 0.4; power, 0.8; alpha level, 0.05) [13], using G*power 3. Statistical significance was set at a *p*-value < 0.05.

## 3. Results

A significant interaction between condition and time was observed for RF stiffness (*p* < 0.05, η_p_^2^ = 0.20). Post hoc tests revealed that RF stiffness significantly decreased post 1 and post 2 compared to pre-intervention (*p* < 0.05; 1 set: d = 0.89; 2 sets: d = 0.74), but no significant difference was observed between post 1 and post 2 (Table 1). On the control side, none of the RF stiffness values changed significantly (*p* > 0.05).

A main effect of time was observed in HBD (*p* < 0.05, η_p_^2^ = 0.45), which changed significantly over time in the passive joint movement condition (*p* < 0.05, d = 0.87), but not in the control condition (*p* > 0.05) (Table 1).

## 4. Discussion

In this study, RF stiffness in patients with OSD decreased after both one and two sets of passive joint movements compared with pre-intervention values; however, no difference was observed between RF stiffness after one and two sets. In addition, the HBD decreased after the two sets compared to the pre-intervention values.

RF stiffness decreased after both one and two sets of passive joint movements compared with pre-intervention values, and HBD improved after two sets in this study. These findings are consistent with the results of a previous study [5]. A review article suggested that reductions in MTU stiffness increase extensibility, thereby improving joint range of motion [7]. The findings of the present study indicate that passive joint movement of the hip and knee in patients with OSD decreases target muscle stiffness and improves HBD test outcomes. However, in a previous study [5], participants performed standing knee-flexion movements, during which subjective pain was assessed. As this procedure might elicit recently alleviated pain, we limited our assessment in the present study to the HBD. The HBD test was conducted by passively flexing the knee to the tolerable limit of the participant; we thus considered it to function as an additional indicator of pain.

The present study demonstrated that RF stiffness in patients with OSD decreased after both one and two sets of passive joint movements, with no significant difference between the two time points. This result suggests that the reduction in RF stiffness may plateau after a single 10 min set of passive joint movements. Continuous mechanical stimulation of muscles through physical therapies such as static stretching induces viscoelastic changes (i.e., a reduction in stiffness) [14]. However, such adaptations of soft tissues slow down once a certain threshold is exceeded due to structural and neural constraints; thus, when static stretching sets are repeated and the duration is prolonged, changes in MTU stiffness and joint flexibility influenced by MTU stiffness eventually stagnate [8,9,10]. Accordingly, the present findings suggest that mechanical stimulation of the RF through passive joint movement induces viscoelastic adaptation, which begins to decelerate within 10 min. Future studies should investigate whether shorter durations of passive joint movement are sufficient to reach this plateau, thereby further optimizing treatment protocols and improving their clinical applicability.

This study had several limitations. First, surface electromyography was not performed; therefore, the level of muscle activity during the RF stiffness measurements was uncertain. To minimize the influence of muscle contraction, the participants were instructed to remain relaxed during the measurements. Second, although the sample size was sufficient for statistical analysis, the small number of female participants may have influenced the results. Third, a previous study [5] stratified patients with OSD by baseline RF stiffness and found that those with higher stiffness showed greater reductions following passive joint movement than those with lower stiffness. The patients with OSD in the present study had baseline RF stiffness comparable to that of the low-stiffness group in a previous study. Consequently, in patients with more severe OSD symptoms and higher RF stiffness, the outcomes may differ, with two sets of passive joint movements (a total of 20 min) potentially producing greater reductions in stiffness than a single 10 min set. Clinically, this suggests that tailoring passive joint movement duration to baseline stiffness levels could optimize therapeutic outcomes and highlights the need for future studies to examine whether patients with higher RF stiffness benefit from extended or repeated passive joint movement protocols.

The RF stiffness observed in this patient cohort was comparable to that in a previously described low-value group [5]. Consequently, our sample might not include patients who would have been classified in the high-value group in that study. We hypothesize that increasing the sample size to include individuals with more pronounced RF stiffness might yield more accurate and reliable results. Furthermore, in other forms of manual therapy, such as muscle stretching, both acute and long-term effects (exceeding 10 weeks) have been investigated [7]. Although in the present study, we examined only acute effects, passive joint movement is often administered over several weeks to months. Therefore, evaluating its long-term follow-up effects is necessary to determine intervention sustainability and enhance clinical relevance. Moreover, given that various patients with OSD are young athletes, investigating whether passive joint movement influences outcomes relevant to return-to-sport decision-making, such as muscle strength and physical function test performance, might be relevant. Further investigation is warranted to determine whether the effects of passive joint movement differ according to individual characteristics, including sex and sport- or position-specific demands such as the repeated transitions from deep knee flexion to a rapid throwing posture in baseball catchers or long-kick actions requiring substantial hip extension and knee flexion in soccer players.

## 5. Conclusions

The results of this study suggest that passive joint movement beyond one set (10 min) might not lead to a further decrease in RF stiffness in the immediate term, suggesting that a second 10 min set might provide only limited additional immediate benefit for reducing RF stiffness in patients with OSD. Such a lack of meaningful difference between a single and multiple sets highlights the time efficiency of the protocol, representing a clinically relevant result for patients with OSD. Future studies should recruit individuals with higher RF stiffness and evaluate long-term and return-to-sport-related outcomes to clarify the durability and clinical relevance of passive joint movement.

## Figures and Tables

**Table 1:** Changes in the shear modulus of the RF and HBD under each condition.

	Passive Joint Movement			Control		
	Pre	Post-1	Post-2	Pre	Post-1	Post-2
RF shear modulus (kPa)	18.9 ± 4.0	17.4 ± 3.3 *	17.3 ± 3.4 *	17.4 ± 4.4	17.8 ± 4.6	17.2 ± 5.0
HBD (cm)	17.0 ± 9.4		13.8 ± 7.2 *	15.4 ± 7.9		14.7 ± 7.4

* Significantly changed compared to pre-intervention (*p* < 0.05). Values are expressed as mean ± standard deviation. RF: Rectus Femoris; HBD: heel–buttock distance.

## Data Availability

The original contributions presented in the study are included in the article, further inquiries can be directed to the corresponding author.
